# COVID-19 vaccine hesitancy in a small rural southern state: Results of a weighted random sample survey

**DOI:** 10.1016/j.heliyon.2024.e40423

**Published:** 2024-11-14

**Authors:** Mark L. Williams, James P. Selig, Benjamin C. Amick III, Ji Li, Don E. Willis, Shashank Kraleti, Sandra M. Meredith-Neve, Sheena CarlLee, Pearl A. McElfish

**Affiliations:** a Fay W. Boozman College of Public Health, University of Arkansas for Medical Sciences, 4301 W. Markham St., Little Rock, AR, 72205, USA; b Fay W. Boozman College of Public Health, University of Arkansas for Medical Sciences Northwest, 2708 S. 48th St., Springdale, AR, 72762, USA; c College of Medicine, University of Arkansas for Medical Sciences Northwest, 2708 S. 48th St., Springdale, AR, 72762, USA; d College of Medicine, University of Arkansas for Medical Sciences, 4301 W. Markham St., Little Rock, AR, 72205, USA; e Integrated Medicine Service Line, University of Arkansas for Medical Sciences, 4301 W. Markham St., Little Rock, AR, 72205, USA; f College of Medicine, University of Arkansas for Medical Sciences Northwest, 1125 N. College Ave., Fayetteville, AR, 72703, USA

**Keywords:** COVID-19, COVID-19 vaccine, Vaccine hesitancy, Rural populations, United States, Arkansas

## Abstract

**Objective:**

COVID-19 remains a significant health threat to the United States (U.S.) and the world even after the development of effective vaccines. Throughout the pandemic, rural areas have been more hesitant to be vaccinated for COVID-19 than more urban counterparts. The purpose of this study was to assess predictors of vaccine hesitancy in a small, largely rural, southern state using a multivariate model that would allow an assessment of independent predictors of COVID-19 vaccine hesitancy.

**Methods:**

Cross-sectional survey research was conducted using data collected from a weighted random sample of 1500 individuals residing in Arkansas, U.S. Data were collected using random digit dialing of landline and cell phone numbers between July 12 and July 30, 2021. Participants were asked about their hesitancy to be vaccinated for COVID-19. Data were analyzed using bivariate odds ratios and cumulative logit model.

**Results:**

Results found 47 % of the sample were hesitant to be vaccinated. Bivariate analysis found vaccine hesitancy to be related to age, race/ethnicity, rural residence, education, employment, and having had COVID-19. Multivariate analysis showed sex, age, rural residence, and history of having had COVID-19 were associated with hesitancy. A history of vaccination/trust in vaccines remained strongly negatively associated with hesitancy.

**Conclusion:**

Findings suggest population diversity may account for vaccine hesitancy across jurisdictions, suggesting public health messages be tailored to a local audience. The strongest finding suggests vaccinations should be emphasized in general to build habits of being vaccinated.

## Highlights

• History of vaccination & trust in vaccines was negatively associated with hesitancy.
• Sex, age, rural residence, & history of COVID-19 infection were associated with hesitancy.
• Population diversity may account for vaccine hesitancy across jurisdictions.
• Public health messages should be tailored to a local audience.
• Vaccinations should be emphasized in general to build habits of being vaccinated.

## 1 Introduction

COVID-19 disease remains a significant health threat to the United States (U.S.) and the entire world. As of September 2022, nearly 95 million Americans have been infected with COVID-19, and more than one million have died because of the virus [1]. Since late November 2020, the serious consequences of COVID-19 disease have been preventable. Three COVID-19 vaccines are currently approved for all Americans, and the vaccines are available free of charge. However, more than a quarter of Americans eligible to be vaccinated are not. At the time of this study (July 2021), just over half of Arkansas adults had received at least one dose of the COVID-19 vaccine [2] As the U.S. enters the third year of the pandemic, with the emergence of new and potentially highly infectious viral variants, fully vaccinating the population for COVID-19 remains a high national priority. However, as we have learned from flu vaccine uptake [3,4], obtaining full population vaccine coverage is not an easy proposition.

Vaccine hesitancy was an issue long before the COVID-19 pandemic, traceable back to the introduction of the smallpox vaccine [5]. Reluctance or refusal to be vaccinated, referred to as vaccine hesitancy [[6], [7], [8]], is highly complex and may apply to some vaccines but not all [7,9]. Personal characteristics found to be associated with vaccine hesitancy are sex, race/ethnicity, education, health literacy, and personal political beliefs [6,[10], [11], [12], [13], [14], [15], [16]]. A person's social network can influence their vaccine hesitancy. People are less hesitant to be vaccinated for COVID-19 if they know someone in their personal network who has been ill or hospitalized with or died of COVID-19 disease [17]. Perhaps the most important factor influencing vaccine hesitancy is a personal history of vaccination. Those vaccinated against one disease are less likely to be hesitant to be vaccinated against another [3,18].

Some factors influencing vaccine hesitancy can be thought of as bridging personal and contextual factors, including lack of medical insurance and access to primary care services [6,19]. These are personal factors in that one can “choose” not to have health insurance or not to visit a primary care provider [20], but they are also strongly influenced by where one lives. People living in rural areas, especially in the Southern U.S., have less opportunity to access health information and medical services, including vaccinations [4,6,21,22]. Rural residents tend to be culturally conservative and less trustful of government and science [23,24]. Lack of access to healthcare and a personal healthcare provider in rural areas may influence whether some are vaccinated for COVID-19. A recommendation for a vaccination from a personal healthcare provider has been found to increase the likelihood of vaccine acceptance [25,26].

Vaccine hesitancy is a context-specific, complex attitude ranging from complete willingness to be vaccinated to a determination to avoid vaccination [27]. Hesitancy to be vaccinated for COVID-19 is unique from hesitancy to be vaccinated for other diseases, such as the flu, although it may also have some similarities. Hesitancy and its predictors are often dependent on specific vaccines [28]. The COVID-19 vaccine was developed under unprecedented social and political conditions, including a global pandemic, increased political polarization, and massive public investment in its development [29]. Furthermore, many of the factors identified as related to COVID-19 vaccine hesitancy have a high degree of collinearity. For example, states with large rural populations, lower educational attainment, lack of insurance, and lack of access to healthcare have consistently been more resistant to COVID-19 vaccination than more urban areas. These factors are also likely highly intercorrelated. The purpose of this study was to assess the predictors of vaccine hesitancy in a small, largely rural, southern state using a multivariate model that would allow an assessment of independent predictors of COVID-19 vaccine hesitancy.

## 2 Methods

### 2.1 Procedures

Data for the cross-sectional survey study were collected from a weighted random sample of Arkansas residents between July 12 and July 30, 2021. Sampling and data collection were completed by Dynata, a national company with experience conducting phone surveys in Arkansas. Dynata also provided survey weights based on 2019 American Community Survey population estimates of race/ethnicity, age, and gender in Arkansas. Participants were contacted using random digit dialing of mixed landline and cell phone numbers. There was a 20 % completion rate for the survey (% who were successfully contacted that completed the survey). The sample was composed of 20 % landline and 80 % cell phone numbers. This ratio was determined based on National Center for Health Statistics state-level estimates of wireless and landline use for Arkansas, which estimate that nearly 80 % of Arkansans used cell phones only or mostly in 2018. Landlines were randomly sampled from a list of all known landlines in the state. Cell phone numbers were randomly selected from a list of cell numbers used in Arkansas. So that larger numbers of Black/African American and Hispanic/Latino residents would be included in the sample, phones in geographic areas with large percentages of minority residents and phones associated with Hispanic/Latino surnames were oversampled.

Trained interviewers called potential participants using a list of phone numbers. If an eligible person answered, the interviewer explained the purpose of the call and asked if he or she was interested in answering a few questions about the COVID-19 pandemic. If the person refused the invitation, the interviewer thanked him/her and proceeded to the next phone number on the list. In the event the person reached spoke only Spanish, an interviewer fluent in Spanish would take over the call. If a person was agreeable, the interviewer obtained verbal informed consent and proceeded to the study questionnaire. Of the people who were successfully contacted, 20 % completed the survey. Given the evidence demonstrating that vaccine hesitancy is present even among the recently vaccinated [30], we did not exclude vaccinated individuals from our analysis. Leading scholars in the field of vaccine hesitancy have argued strongly for a definition which distinguishes it from the behavior of vaccination and allows for the possibility of indecisiveness even among the vaccinated [31,32]. Moreover, ignoring vaccine hesitancy among those who receive a vaccine dose may prove unwise given that hesitancy could lead to a different decision for subsequent doses, not to mention inaccurate estimations of vaccine hesitancy in any given population.

Study procedures and materials were reviewed by the University of Arkansas for Medical Sciences Institutional Review Board (IRB) and determined to be exempt on June 14, 2021 (IRB #262907). Documentation of consent was not required. Instead, participants indicated consent by agreeing to complete the survey after the study was explained to them.

### 2.2 Eligibility

Eligible individuals who participated in the study were: 18 years or older; a resident of Arkansas; and able to understand and speak English or Spanish. The study was explained to the listener, and s/he was asked if s/he was willing to proceed with the survey. Willingness to go forward with the survey signified consent.

### 2.3 COVID-19 vaccine hesitancy

Vaccine hesitancy, the dependent factor, was measured using a single item: “Thinking specifically about COVID-19 vaccines, how hesitant are/were you about getting vaccinated?” [33]. Responses were recorded using a four-point Likert-type scale ranging from not at all hesitant to very hesitant.

### 2.4 Demographic and other characteristic

Participants were asked to describe themselves using several sociodemographic descriptors, including sex, age, race/ethnicity, education, employment, and residence [34]. Age was measured in years and recoded as: 18 to 29; 30 to 44; 45 to 59; and greater than or equal to 60 [34]. Race/ethnicity was measured as White, Black/African American, Hispanic/Latino, and other [34]. Education was measured as high school (HS) diploma/graduate equivalency degree (GED) or lower, some college, associate degree, and bachelor's degree or higher [34]. Employment was measured as unemployed or student, employed, or retired [34]. Residence was measured by zip code and recoded as urban or rural based on Rural-Urban Commuting Area (RUCA) codes [35].

### 2.5 Health and healthcare

A five-point Likert-type scale ranging from poor to excellent was used to measure participants’ perception of their current health [36,37]. The responses excellent, very good, and good were collapsed to a single category, good or better, resulting in a three-point measurement (i.e., good or better, fair, or poor) [36,37]. Having health insurance was measured as yes/no [34].

Participants were asked if they had a current primary healthcare provider, with yes/no response, and the time since last seeing the provider [38]. Time since last seeing a provider was measured using a four-point Likert scale ranging from never to within the past year, then recoded to within the past 2 years and more than 2 years ago. To minimize the potential for excess collinearity among predictors and build a more parsimonious model, the two conceptually similar and positively correlated items assessing access to healthcare (do you have a current primary healthcare provider and time since last seeing a provider) were combined by averaging the items. Although the intent was not to develop a new scale, the items were examined to insure a positive correlation and an exploratory factor analysis (EFA) showed the items loaded on the same factor (Cronbach's α = .57; M = .86; SD = .29).

### 2.6 Past COVID-19 and COVID-19 in personal network

Participants were asked if they had tested positive for COVID-19 or suspected they had been positive for COVID-19. Participants provided a dichotomous yes/no response. The effect of COVID-19 in one's personal network was measured by asking the participant if s/he had close family or friends who tested positive, or suspected they were positive, or died from the disease. Questions regarding infection were adapted from survey items from an AP-NORC survey, and the question regarding COVID-19 deaths comes directly from it.

### 2.7 History of vaccination and trust in vaccines

Participants were asked to respond to vaccine-related measures other than those associated with COVID-19 [33]. History of vaccination was measured by asking a participant how many times s/he had been vaccinated for the flu in the past five years. Four response choices were possible, ranging from never to every year. Items measuring trust in vaccinations in general and general hesitancy to receive any vaccine were also measured by four-point Likert-type scales. Participants were also asked the proportion of people close to them that have received the COVID-19 vaccine, with four response choices ranging from very few to nearly all. As above, these four conceptually related and positively correlated items (history of vaccination, flu vaccination, trust in vaccines, and general vaccine hesitancy) were combined by averaging the item scores (Cronbach's α = .67; M = 3.08; SD = .74), and EFA results showed the items loaded on the same factor.

### 2.8 Analyses

The study sample included 1500 participants. The percentage of missing values for each variable ranged from zero to 3 %. Participants with incomplete responses (n = 124; 8.3 %) were omitted from the analyses, resulting in a final analytic sample of 1376. Survey weights were used in the analyses to ensure results would generalize to the population.

Bivariate odds ratios were first calculated to discover factors that appeared to have an effect on COVID-19 vaccine hesitancy and eliminate those that did not. Factors showing an effect and their interactions were then tested using a cumulative logit model. The cumulative logit model produces a model of factors having an independent effect on vaccine hesitancy controlling for all other factors entered in the model. Interactions between significant bivariate factors were tested because it was reasonable to assume some measures may have had a multiplicative effect when in the presence of another. The model was structured to predict having higher scores on the ordinal hesitancy measure. The score test for the proportional odds assumption was non-significant (χ^2^ = 51.35 with df = 40; *p* = .11) and consistent with the proportional odds assumption. SAS 9.4 was used for descriptive statistics, bivariate analyses, and ordinal logistic regression analysis. R 4.1.3 was used to test goodness-of-fit of the model [39].

## 3 Results

### 3.1 Sample

More than half of those participating in the study, 53 %, said they were not at all hesitant to receive a COVID-19 vaccine. Slightly more than a quarter said they were either a little or somewhat hesitant, and 21 % said they were very hesitant. As shown in Table 1, most participants were white, female, older than 60, and urban residents. About a third had a high school/GED or less education, and slightly less than a quarter had some college education. One-third of the sample said they lived in a rural area. About half were employed, and a third were retired. More than two-thirds said their health was good or better, and most had health insurance. Almost a quarter of the sample reported having confirmed or suspected COVID-19, with three quarters saying they had a family member or friend who had suspected or tested positive for COVID-19 or had died from the disease.

**Table 1:** Odds of being COVID-19 vaccine hesitant.

	Unweighted n (%)	Odds Ratio	95 % CI	
Sex				
Male	598(40 %)	–	–	–
Female	893(60 %)	1.01	.83	1.22
**Age**				
18–29	228(16 %)	**2.59**	1.97	3.42
30–44	257(18 %)	**2.57**	1.98	3.35
45–59	316(22 %)	**2.05**	1.55	2.66
>60+	653(45 %)	–	–	–
**Race/ethnicity**				
White	757(50 %)	–	–	–
Black/African American	366(24 %)	1.20	.92	1.57
Hispanic/Latino	253(17 %)	**1.44**	1.01	2.05
Other	124(8 %)	1.48	.96	2.28
**Education**				
< HS/GED	480(32 %)	**2.60**	2.05	3.31
Some college	337(23 %)	**2.07**	1.60	2.69
Associate degree	159(11 %)	**2.14**	1.53	3.00
> Bachelor's degree or higher	517(35 %)	–	–	–
**Employment**				
Unemployed/student	289(19 %)	–	–	–
Employed	716(48 %)	.87	.68	1.11
Retired	483(32 %)	**.34**	.26	.47
**Residence**				
Urban	1029(69 %)	–	–	–
Rural	471(31 %)	**1.46**	1.19	1.79
**Health**				
Good or better	164(70 %)	–	–	–
Fair	328(22 %)	.94	.73	1.20
Poor	128(9 %)	1.17	.83	1.65
**Insurance coverage**				
No	165(11 %)	**2.17**	1.62	2.91
Yes	1320(89 %)	–	–	–
**Access to healthcare**	–	**.38**	.28	.51
**Had COVID-19**				
No	1135(76 %)	–	–	–
Yes	356(24 %)	**1.86**	1.49	2.31
**Family/friends had COVID-19**				
No	406(27 %)	–	–	–
Yes	1073(73 %)	1.11	.89	1.38
**History of/trust in vaccines**	–	**.12**	.10	.15

Note: Bolded odds ratios represent their *p*-value <.05. CI = confidence interval; GED = graduate equivalency degree; HS = high school.

### 3.2 Hesitancy

Compared to those age 60 or older, younger participants had significantly greater odds of being hesitant to receive the COVID-19 vaccine. Hispanic/Latino participants had significantly higher odds of being hesitant than white participants. Rural residents had significantly higher odds of hesitancy than urban residents. Lower levels of education were associated with significantly higher odds of hesitancy. Those with a high school diploma/GED or lower and some college or associate degree had two to three times greater odds of being COVID-19 vaccine hesitant compared to those with a bachelor's degree or higher.

Compared to participants who were unemployed or students, those who were retired had significantly lower odds of being vaccine hesitant. Participants who reported a history of confirmed or suspected COVID-19 had significantly higher odds of hesitancy than those not reporting confirmed or suspected COVID-19. The odds of being vaccine hesitant for participants with no healthcare insurance coverage were more than twice that of those with insurance. The composite measures, history of/trust in vaccines and access to healthcare, were significantly associated with decreased vaccine hesitancy.

### 3.3 Multivariable model

All factors were entered into a multivariable cumulative logit model, as shown in Table 2. Five were found to significantly predict COVID-19 vaccine hesitancy. Four predictors showed increased odds of vaccine hesitancy, while one showed decreased odds. Unlike the bivariate results, sex emerged as a significant predictor in the multivariate model. Female participants had approximately 1.5 greater odds (*OR* = 1.55; 95 % *CI* 1.22–1.95; *p* < .001) of being vaccine hesitant than male participants. Other positive predictors of vaccine hesitancy were being age 30 to 44 (vs. >60+; *OR* = 1.69; 95 % *CI* 1.13–2.52; *p=*.01), residing in a rural area (vs. urban; *OR* = 1.35; 95 % *CI* 1.06–1.73; *p=*.02), and having a history of confirmed or suspected COVID-19 infection (vs. no; *OR* = 1.98; 95 % *CI* 1.52–2.58; *p* < .001). The strongest predictor of vaccine hesitancy, using the beta weight as the measure, was negative. A one unit increase in the history of/trust in vaccination composite score was associated with a decrease in the odds of hesitancy by a factor of .12 (*OR* = .12; 95 % *CI* .10-.15; *p* < .001). Race/ethnicity, education, employment, access to healthcare, and insurance coverage, which showed bivariate effects, were not found to significantly predict COVID-19 vaccine hesitancy when controlling for other factors entered into the model. An interaction between sex and other independent factors was investigated, and no interaction was found.

**Table 2:** Weighted ordinal logistic regression – COVID-19 Vaccination hesitancy.

	*B*	*SE*	*p*	*OR*		*95 % CI*
Sex						
Male	–	–	–	–	–	–
Female	**.435**	**.119**	**< .001**	**1.55**	**1.22**	**1.95**
**Age**						
18–29	.400	.219	.068	1.49	.97	2.29
30–44	**.525**	**.204**	**.010**	**1.69**	**1.13**	**2.52**
45–59	.299	.195	.125	1.35	.92	1.98
≥60+	–	–	–	–	–	–
**Race/ethnicity**						
White	–	–	–	–	–	–
Black/African American	−.285	.162	.079	.75	.55	1.03
Hispanic/Latino	.129	.214	.547	1.14	.75	1.73
Other	−.111	.257	.664	.90	.54	1.48
**Education**						
≤ HS/GED	−.018	.158	.911	.98	.72	1.34
Some college	.155	.160	.331	1.17	.85	1.60
Associate degree	.225	.194	.247	1.25	.86	1.83
≥ Bachelor's degree	–	–	–	–	–	–
**Employment**						
Unemployed/student	–	–	–	–	–	–
Employed	−.093	.156	.551	.91	.67	1.24
Retired	−.244	.225	.277	.78	.51	1.28
**Residence**						
Urban	–	–	–	–	–	–
Rural	**.303**	**.125**	**.016**	**1.35**	**1.06**	**1.73**
**Health**						
Good or better	–	–	–	–	–	–
Fair	−.140	.155	.368	.87	.64	1.18
Poor	.162	.221	.465	1.18	.76	1.82
**Insurance coverage**						
Yes	–	–	–	–	–	–
No	.171	.177	.333	1.19	.84	1.68
**Access to healthcare**	**.390**	**.202**	**.053**	**1.48**	**1.00**	**2.19**
**Had COVID-19**						
No	–	–	–	–	–	–
Yes	**.684**	**.134**	**<.001**	**1.98**	**1.52**	**2.58**
**Family/friends had COVID-19**						
No	–	–	–	–	–	–
Yes	−.105	.139	.449	.90	.69	1.18
**History of/trust in vaccines**	**−2.13**	**.103**	**<.001**	**.12**	**.10**	**.15**

Note: Reference group same as bivariate odds. CI = confidence interval; GED = graduate equivalency degree; HS = high school; OR = odds ratio; SE = standard error.

Following guidance from Fagerland and Hosmer [40], the predictive model's fit was assessed using three goodness of fit tests, Hosmer-Lemeshow, Lipsitz, and Pulkstenis-Robinson. Both the Lipsitz (p = . 06) and Pulkstenis-Robinson (p = .99) tests were not significant, suggesting adequate model fit. The Hosmer-Lemeshow test (p = .01), however, indicated inadequate fit. The addition of a two-way interaction between sex and race/ethnicity resulted in acceptable model fit (Hosmer-Lemeshow p = .17; Lipsitz p = .10; and Pulkstenis-Robinson p = .99), but the interaction was not found to be statistically significant.

## 4 Discussion

This study was conducted to assess the predictors of vaccine hesitancy in a small, largely rural, southern state. The study found that a quarter of participants in this weighted random sample of residents of a rural southern state were very hesitant to be vaccinated for COVID-19, which is consistent with data from another national study [12]. Being female, living in a rural area, and having a history of vaccination were found to be predictors of COVID-19 vaccine hesitancy. More than half reported no hesitancy about being vaccinated.

Like other studies, this study found that residing in a rural area predicted increased vaccine hesitancy [6,21]. Rurality is associated with low health literacy, conservative political views, and closed community networks. Low health literacy suggests lower awareness and understanding of health, preventative health measures, and vaccines in particular [14,22,41]. Rural areas of the U.S., especially those in conservative states, have tended to spurn not only COVID-19 vaccines, but also other COVID-19 preventive measures, such as mask wearing and social distancing. This may in large part be due to the unfortunate politicization of the pandemic and COVID-19 preventative measures [42,43]. Social networks in rural areas may also be influential, especially if those networks are closed. Social networks strongly influence the formation and reinforcement of normative behaviors and can inhibit seeking out new information. The social networks of rural residents are likely to be made up of others who reside in the same area and share the same views and behaviors. If those views are strongly vaccine hesitant, network members would be less likely to encounter contrary information or behaviors that would encourage vaccination. Having had COVID-19 was found to be predictive of increased vaccine hesitancy. Those who have had COVID-19 may be convinced they do not need to be vaccinated.

The strongest predictor of vaccine hesitancy was the measure of past vaccine history and trust in vaccines [4,18,44,45]. This finding may have important implications for developing a trust in vaccines, including COVID-19. Greater experience with vaccinations may lead to growing trust in vaccines in general and, eventually, the development of vaccination as a habitual behavior requiring little thought. Parents may pass this behavior on to their children as they demonstrate vaccination behavior both by being vaccinated and having their children vaccinated. To increase experience with vaccines and vaccinations, young parents should receive education about vaccinating their young children, and education should be reinforced by healthcare workers and others providing public health services. Parents participating in birthing classes should be provided with information about vaccinating their children throughout their lifetimes. Public and private programs providing services to pre- and postnatal women should include educational components focusing on the importance of vaccinations for the health of their children. Others not involved with these types of programs should receive information about vaccines and vaccinations from their pediatric healthcare providers.

This study is not without limitations. While findings are based on a weighted random sample of a statewide population, they may not be generalizable to other states or non-U.S. populations, although rates of vaccine hesitancy are like those found in other studies [26]. Populations without landlines or cell phones are excluded from random digit dialing methods. Results may be further limited by the sample being older, more female, more educated, and slightly wealthier than the state's residents. This may be due to the time of day when data were collected and the willingness of those most likely to answer a phone call. While it is a limitation, it may have provided clearer distinctions between those who were COVID-19 vaccine hesitant and those who were not. Responses to survey questions were self-reported and are subject to the biases associated with self-report measures. However, given the anonymous nature of the survey, there is little reason to suspect participants were inclined to be dishonest or devious when responding to survey items. More likely, those who were inclined to be secretive about their COVID-19 opinions or suspicious of a survey did not participate. The study was also limited because it used a single measure of COVID-19 vaccine hesitancy, where a composite measure may have more accurately reflected the complexity of vaccine hesitancy [46].

Despite these limitations, this study's findings are useful. Findings suggest public health programs developed using national or even state level data may not adequately account for population diversity. Rural areas are most likely to have different vaccine dynamics than urban areas, particularly in the more traditional southern states. Some of this difference may be related to health literacy, but certainly conservatism plays a large role [10,44]. This study and others strongly suggest public health messages must be tailored to a local audience. COVID-19 vaccination remains an important public health measure. Findings also strongly suggest outreach programs to encourage vaccinations cannot be a singular effort tied to encouraging vaccination for a specific disease. Rather, programs to encourage vaccination must be long term and encourage vaccination in general. In this way, vaccination for a new disease may be a matter of habit, rather than a new behavior. As the COVID-19 pandemic continues, the issue of vaccine hesitancy remains important. The COVID-19 virus has a great capacity to mutate [47], and current medications may be of limited use over the long term [48]. As the pandemic continues, vaccines and boosters will remain necessary for maintaining the public's health.

## CRediT authorship contribution statement

**Mark L. Williams:** Writing – review & editing, Writing – original draft, Formal analysis, Conceptualization. **James P. Selig:** Writing – review & editing, Writing – original draft, Formal analysis, Conceptualization. **Benjamin C. Amick III:** Writing – review & editing, Writing – original draft. **Ji Li:** Formal analysis. **Don E. Willis:** Writing – review & editing, Writing – original draft, Formal analysis, Conceptualization. **Shashank Kraleti:** Writing – review & editing, Writing – original draft. **Sandra M. Meredith-Neve:** Writing – review & editing, Writing – original draft. **Sheena CarlLee:** Writing – review & editing, Writing – original draft. **Pearl A. McElfish:** Writing – review & editing, Writing – original draft, Conceptualization.

## Ethics statement

Study procedures and materials were reviewed by the University of Arkansas for Medical Sciences Institutional Review Board (IRB) and determined to be exempt on June 14, 2021 (IRB #262907). Documentation of consent was not required. Instead, participants indicated consent by agreeing to complete the survey after the study was explained to them.

## Data availability

The deidentified data underlying the results presented in this study may be made available upon request from the corresponding author, Dr. Pearl A. McElfish, at pamcelfish@uams.edu. The data are not publicly available in accordance with funding requirements and participant privacy.

## Funding

This work is supported by 10.13039/100008519University of Arkansas for Medical Sciences 10.13039/501100020492Translational Research Institute funding awarded through the 10.13039/100006108National Center for Advancing Translational Sciences of the 10.13039/100000002National Institutes of Health (10.13039/100000002NIH) (UL1 TR003107); Community Engagement Alliance (CEAL) Against COVID-19 Disparities (10.13039/100000002NIH 10T2HL156812-01); and Rapid Acceleration of Diagnostics (RADx) (10.13039/100000002NIH 3 R01MD013852-03S2). The content is solely the responsibility of the authors and does not necessarily represent the official views of the funders.

## Declaration of competing interest

The authors declare the following financial interests/personal relationships which may be considered as potential competing interests:

Dr. Sheena CarlLee reports owning some Pfizer stock. All other authors declared no conflicts of interest.
