# Supplementary material for: COVID-19 vaccine hesitancy in a small rural southern state: Results of a weighted random sample survey
Source: Heliyon. 2024 Nov 14;10(22):e40423. doi: 10.1016/j.heliyon.2024.e40423 (PMC11617713; doi:10.1016/j.heliyon.2024.e40423)
Supplement: Multimedia component 1 [file mmc1.docx]

-------------------------------------------------------------------------

**AR POLITICAL ISSUES DATA MAP**

-------------------------------------------------------------------------

Question: qintro - 1 (Single)

Text:

Hello, My name is . I'm calling on behalf of University of Arkansas for Medical Sciences to invite you to volunteer in a study involving research on health, vaccines, and social life. Participation involves answering survey questions that will take approximately 10 minutes of your time. This is completely voluntary. If you agree to participate, you have the right to answer only the questions you want to answer and end participation at any point. Potential risks of this research are minimal and your answers will remain confidential and only the investigators and study staff will see the data-we will not ask your name, or address. Nothing about your medical care will change as a result of the decision you make. By completing this survey, you are consenting to collect your survey responses.

Do you agree to participate in the survey?

Participant Rights: If you have any questions regarding your rights as a participant in this research and/or concerns about the study, or if you feel under any pressure to enroll or to continue to participate in this study, you may contact the University of Arkansas for Medical Sciences Institutional Review Board (which is a group of people who review the research studies to protect participants' rights) at (501) 686-5667 or IRB@uams.edu or the principal investigator, Pearl McElfish at (479) 713-8680.

(qintro:1)Continue

(qintro:2)Callback

(qintro:3)Refused

-------------------------------------------------------------------------

Question: q1 - 1 (Single)

Text:

Do you live in Arkansas?

(q1:1)Yes

(q1:2)No

(q1:3)Refused

-------------------------------------------------------------------------

Question: q2 - 1 (Single)

Text:

Are you 18 years of age or older?

(q2:1)Yes - Male [Interviewer assumes respondent is a man]

(q2:2)Yes - Female [Interviewer assumes respondent is a woman]

(q2:3)No - Thank you very much but we are only interviewing person age 18 or older at this time.

(q2:4)Refused

-------------------------------------------------------------------------

Question: q51 - 1 (Single)

Text:

Are you Hispanic, Latino/a, or Spanish origin?

(q51:1)Yes

(q51:2)No

(q51:3)Don't know / not sure

(q51:4)Refused

-------------------------------------------------------------------------

Question: q51a - 7 (Multiple)

Text:

If yes selected, read and code subcategories underneath major heading. One or more categories may be selected.

(q51a:1)Mexican, Mexican American, Chicano/a

(q51a:2)Puerto Rican

(q51a:3)Cuban

(q51a:4)Another Hispanic, Latino/a, or Spanish origin

-------------------------------------------------------------------------

Question: q52 - 17 (Multiple)

Text:

Which one or more of the following would you say is your race?

(q52:1)White

(q52:2)Black or African American

(q52:3)American Indian or Alaska Native

(q52:4)Asian

(q52:5)Pacific Islander

(q52:6)Other

(q52:7)No additional choices

(q52:8)Don't know / not sure

(q52:9)Refused

-------------------------------------------------------------------------

Question: q52a - 35 (Multiple)

Text:

If Asian or Pacific Islander are selected, read and code subcategories underneath major heading. One or more categories may be selected.

(q52a:01)Asian Indian

(q52a:02)Chinese

(q52a:03)Filipino

(q52a:04)Japanese

(q52a:05)Korean

(q52a:06)Vietnamese

(q52a:07)Other Asian

(q52a:08)Native Hawaiian

(q52a:09)Guamanian or Chamorro

(q52a:10)Samoan

(q52a:11)Marshallese (NOT IN ORIGINAL BRFSS, appropriate to add because Arkansas is home to the largest population of Marshallese in the continental US)

(q52a:12)Other Pacific Islander

-------------------------------------------------------------------------

Question: qquota - 1 (Single)

Text:

QUOTA RACE

(qquota:1)Hispanic

(qquota:2)White

(qquota:3)AA

(qquota:4)Others

-------------------------------------------------------------------------

Question: q3 - 4 (Numeric)

Text:

What year were you born? - year

-------------------------------------------------------------------------

Question: q3x - 1 (Multiple)

Text:

Exclusive Options: What year were you born?

(q3x:1)Refused

-------------------------------------------------------------------------

Question: q4 - 1 (Single)

Text:

Are you the parent or guardian of a child under age 18?

(q4:1)Yes

(q4:2)No

(q4:3)Refused

-------------------------------------------------------------------------

Question: q5 - 7 (Multiple)

Text:

Are(Is) your children(child) 0-4 years old, 5-11 years old, or 12-17 years old?

(q5:1)0-4 years old

(q5:2)5-11 years old

(q5:3)12-17 years old

(q5:4)Refused

-------------------------------------------------------------------------

Question: q6 - 1 (Single)

Text:

Have you received a COVID-19 Vaccine?

(q6:1)Yes

(q6:2)No

(q6:3)Don't know / not sure

(q6:4)Refused

-------------------------------------------------------------------------

Question: q7 - 1 (Single)

Text:

Did you receive (or do you plan to receive) all required doses?

(q7:1)Yes

(q7:2)No

(q7:3)Don't know / not sure

(q7:4)Refused

-------------------------------------------------------------------------

Question: q8 - 1 (Single)

Text:

Are you planning to...

(q8:1)Definitely get a vaccine

(q8:2)Probably get a vaccine

(q8:3)Be unsure about getting a vaccine

(q8:4)Probably NOT get a vaccine

(q8:5)Definitely NOT get a vaccine

(q8:6)Refused

-------------------------------------------------------------------------

Question: q9 - 32 (Multiple)

Text:

Which of the following, if any, are reasons that you ? Please tell me all that apply.

(q9:01)I am concerned about possible side effects of a COVID-19 vaccine

(q9:02)I don't know if a COVID-19 vaccine will work

(q9:03)I don't believe I need a COVID-19 vaccine

(q9:04)I don't like vaccines

(q9:05)My doctor has not recommended it

(q9:06)I plan to wait and see if it is safe and may get it later

(q9:07)I think other people need it more than I do right now

(q9:08)I am concerned about the cost of a COVID-19 vaccine

(q9:09)I don't trust COVID-19 vaccines

(q9:10)I don't trust the government

(q9:11)Other (please specify)

-------------------------------------------------------------------------

Question: q9oth1 - 50 (Open-end)

Text:

Which of the following, if any, are reasons that you ? Please tell me all that apply. - Other

-------------------------------------------------------------------------

Question: q10 - 1 (Single)

Text:

Once there is a COVID-19 vaccine authorized and available for your child's age group, do you think you will...

(q10:1)Get them vaccinated right away

(q10:2)Wait a while to see how it is working

(q10:3)Only get your child vaccinated if their school requires it

(q10:4)Definitely not get them vaccinated

(q10:5)Child is already vaccinated (if volunteered as a response)

(q10:6)Refused

-------------------------------------------------------------------------

Question: q11 - 1 (Single)

Text:

Regarding a COVID-19 vaccine for your child, do you think you will...

(q11:1)Get them vaccinated right away

(q11:2)Wait a while to see how it is working

(q11:3)Only get your child vaccinated if their school requires it

(q11:4)Definitely not get them vaccinated

(q11:5)Child is already vaccinated (if volunteered as a response)

(q11:6)Refused

-------------------------------------------------------------------------

Question: q12 - 1 (Single)

Text:

How many years in past 5 years have you gotten a seasonal flu vaccine?

(q12:1)Never

(q12:2)1-2 years

(q12:3)3-4 years

(q12:4)Every year

(q12:5)Don't know / not sure

(q12:6)Refused

-------------------------------------------------------------------------

Question: q16 - 1 (Single)

Text:

Overall, how hesitant are you about getting vaccinations? Would you say..

(q16:1)Not at all hesitant

(q16:2)A little hesitant

(q16:3)Somewhat hesitant

(q16:4)Very hesitant

-------------------------------------------------------------------------

Question: q17 - 1 (Single)

Text:

Overall, how much do you trust vaccines?

(q17:1)Not at all

(q17:2)Very little

(q17:3)Somewhat

(q17:4)Very much

-------------------------------------------------------------------------

Question: q18 - 1 (Single)

Text:

Thinking specifically about the COVID-19 vaccines, how hesitant are/were you about getting vaccinated? Would you say..

(q18:1)Not at all hesitant

(q18:2)A little hesitant

(q18:3)Somewhat hesitant

(q18:4)Very hesitant

-------------------------------------------------------------------------

Question: q19 - 1 (Single)

Text:

How much do you trust the COVID-19 vaccines? Would you say..

(q19:1)Not at all

(q19:2)Very little

(q19:3)Somewhat

(q19:4)Very much

-------------------------------------------------------------------------

Question: q20 - 1 (Single)

Text:

Of the people close to you, what proportion do you think have received the COVID-19 vaccine? Would you say...

(q20:1)Very few

(q20:2)Some, but not many

(q20:3)Many

(q20:4)Nearly all

-------------------------------------------------------------------------

Question: q22 - 1 (Single)

Text:

How do other people usually classify you in this country? Would you say: White, Black or African American, Hispanic or Latino, Asian, Native Hawaiian or other Pacific Islander, American Indian or Alaskan Native, or some other group? We want to know how OTHER people usually classify you in this country, which might be different from how you classify yourself.

(q22:1)White

(q22:2)Black or African American

(q22:3)Hispanic or Latino

(q22:4)Asian

(q22:5)Native Hawaiian or Other Pacific Islander

(q22:6)American Indian or Alaska Native

(q22:7)Some other group (please specify)

(q22:8)Don't know / not sure

(q22:9)Refused

-------------------------------------------------------------------------

Question: q22oth1 - 50 (Open-end)

Text:

How do other people usually classify you in this country? Would you say: White, Black or African American, Hispanic or Latino, Asian, Native Hawaiian or other Pacific Islander, American Indian or Alaskan Native, or some other group? We want to know how OTHER people usually classify you in this country, which might be different from how you classify yourself. - Some other group

-------------------------------------------------------------------------

Question: q23 - 1 (Single)

Text:

How often do you think about your race? Would you say...

(q23:1)Never

(q23:2)Once a year

(q23:3)Once a month

(q23:4)Once a week

(q23:5)Once a day

(q23:6)Once an hour

(q23:7)Constantly

(q23:8)Don't know / not sure

(q23:9)Refused

-------------------------------------------------------------------------

Question: q24 - 1 (Single)

Text:

In this next section I am going to ask you how you are treated. Have you ever experienced discrimination, been prevented from doing something, or been hassled or made to feel inferior in any of the following situations because of your race, ethnicity, or color? You can respond to each of these with never, once, two or three times, four or more times. - At school?

(q24:1)Never

(q24:2)Once

(q24:3)Two or three times

(q24:4)Four or more times

-------------------------------------------------------------------------

Question: q25 - 1 (Single)

Text:

In this next section I am going to ask you how you are treated. Have you ever experienced discrimination, been prevented from doing something, or been hassled or made to feel inferior in any of the following situations because of your race, ethnicity, or color? You can respond to each of these with never, once, two or three times, four or more times. - Getting hired or getting a job?

(q25:1)Never

(q25:2)Once

(q25:3)Two or three times

(q25:4)Four or more times

-------------------------------------------------------------------------

Question: q26 - 1 (Single)

Text:

In this next section I am going to ask you how you are treated. Have you ever experienced discrimination, been prevented from doing something, or been hassled or made to feel inferior in any of the following situations because of your race, ethnicity, or color? You can respond to each of these with never, once, two or three times, four or more times. - At work?

(q26:1)Never

(q26:2)Once

(q26:3)Two or three times

(q26:4)Four or more times

-------------------------------------------------------------------------

Question: q27 - 1 (Single)

Text:

In this next section I am going to ask you how you are treated. Have you ever experienced discrimination, been prevented from doing something, or been hassled or made to feel inferior in any of the following situations because of your race, ethnicity, or color? You can respond to each of these with never, once, two or three times, four or more times. - Getting housing?

(q27:1)Never

(q27:2)Once

(q27:3)Two or three times

(q27:4)Four or more times

-------------------------------------------------------------------------

Question: q28 - 1 (Single)

Text:

In this next section I am going to ask you how you are treated. Have you ever experienced discrimination, been prevented from doing something, or been hassled or made to feel inferior in any of the following situations because of your race, ethnicity, or color? You can respond to each of these with never, once, two or three times, four or more times. - Getting medical care?

(q28:1)Never

(q28:2)Once

(q28:3)Two or three times

(q28:4)Four or more times

-------------------------------------------------------------------------

Question: q29 - 1 (Single)

Text:

In this next section I am going to ask you how you are treated. Have you ever experienced discrimination, been prevented from doing something, or been hassled or made to feel inferior in any of the following situations because of your race, ethnicity, or color? You can respond to each of these with never, once, two or three times, four or more times. - Getting service in a store or a restaurant?

(q29:1)Never

(q29:2)Once

(q29:3)Two or three times

(q29:4)Four or more times

-------------------------------------------------------------------------

Question: q30 - 1 (Single)

Text:

In this next section I am going to ask you how you are treated. Have you ever experienced discrimination, been prevented from doing something, or been hassled or made to feel inferior in any of the following situations because of your race, ethnicity, or color? You can respond to each of these with never, once, two or three times, four or more times. - Getting credit, bank loans, or a mortgage?

(q30:1)Never

(q30:2)Once

(q30:3)Two or three times

(q30:4)Four or more times

-------------------------------------------------------------------------

Question: q31 - 1 (Single)

Text:

In this next section I am going to ask you how you are treated. Have you ever experienced discrimination, been prevented from doing something, or been hassled or made to feel inferior in any of the following situations because of your race, ethnicity, or color? You can respond to each of these with never, once, two or three times, four or more times. - On the street or in a public setting?

(q31:1)Never

(q31:2)Once

(q31:3)Two or three times

(q31:4)Four or more times

-------------------------------------------------------------------------

Question: q32 - 1 (Single)

Text:

In this next section I am going to ask you how you are treated. Have you ever experienced discrimination, been prevented from doing something, or been hassled or made to feel inferior in any of the following situations because of your race, ethnicity, or color? You can respond to each of these with never, once, two or three times, four or more times. - From the police or in the courts?

(q32:1)Never

(q32:2)Once

(q32:3)Two or three times

(q32:4)Four or more times

-------------------------------------------------------------------------

Question: q36 - 1 (Single)

Text:

Do you have any kind of health care coverage, including health insurance, prepaid plans, such as HMOs, government plans such as Medicare, or Indian Health Service?

(q36:1)Yes

(q36:2)No

(q36:3)Don't know / not sure

(q36:4)Refused

-------------------------------------------------------------------------

Question: q37 - 1 (Single)

Text:

Do you have one person you think of as your personal doctor or health care provider? Is there more than one, or is there no person you think of as your personal doctor or health care provider?

(q37:1)Yes, only one

(q37:2)More than one

(q37:3)No

(q37:4)Don't know / not sure

(q37:5)Refused

-------------------------------------------------------------------------

Question: q38 - 1 (Single)

Text:

Was there a time in the past 12 months when you needed a doctor but could not see one because of the cost?

(q38:1)Yes

(q38:2)No

(q38:3)Don't know / not sure

(q38:4)Refused

-------------------------------------------------------------------------

Question: q39 - 1 (Single)

Text:

About how long has it been since you last visited a doctor for a routine checkup? A routine checkup is a general physical exam, not an exam for a specific injury, illness, or condition. Would you say?

(q39:1)Within the past year (anytime less than 12 months ago)

(q39:2)Within the past 2 years (1 year but less than 2 years ago)

(q39:3)Within the past 5 years (2 years but less than 5 years ago)

(q39:4)5 or more years ago

(q39:5)Don't know / not sure

(q39:6)Never

(q39:7)Refused

-------------------------------------------------------------------------

Question: q40 - 1 (Single)

Text:

Would you say that in general your health is-

(q40:1)Excellent

(q40:2)Very good

(q40:3)Good

(q40:4)Fair

(q40:5)Poor

(q40:6)Don't know / Not sure

(q40:7)Refused

-------------------------------------------------------------------------

Question: q41 - 1 (Single)

Text:

Have you tested positive for, or suspect that you have had, COVID-19?

(q41:1)Yes - I tested positive for COVID-19

(q41:2)Yes - I suspect that I had COVID-19

(q41:3)No - I do not believe I had COVID-19

(q41:4)Don't know / not sure

-------------------------------------------------------------------------

Question: q42 - 1 (Single)

Text:

Have any close friends or relatives tested positive for, or suspect that they have had, COVID-19?

(q42:1)Yes - they tested positive for COVID-19

(q42:2)Yes - they suspect that they had COVID-19

(q42:3)No - I do not believe any close friends or relatives had COVID-19

(q42:4)Don't know / not sure

(q42:5)Refused

-------------------------------------------------------------------------

Question: q43 - 1 (Single)

Text:

Has a close friend or relative died of COVID-19?

(q43:1)Yes

(q43:2)No

(q43:3)Don't know / not sure

(q43:4)Refused

-------------------------------------------------------------------------

Question: q50 - 1 (Single)

Text:

What is your gender?

(q50:1)Man

(q50:2)Woman

(q50:3)Non-binary

(q50:4)Prefer to self-describe

(q50:5)Refused

-------------------------------------------------------------------------

Question: q50oth1 - 50 (Open-end)

Text:

What is your gender? - Prefer to self-describe

-------------------------------------------------------------------------

Question: q53 - 1 (Single)

Text:

What is your relationship status?

(q53:1)Married

(q53:2)Divorced

(q53:3)Widowed

(q53:4)Separated

(q53:5)Never married

(q53:6)A member of an unmarried couple

(q53:7)Refused

-------------------------------------------------------------------------

Question: q54 - 1 (Single)

Text:

What is the highest degree or level of school you have completed?

(q54:1)Less than high school

(q54:2)Some high school

(q54:3)High school graduate or equivalent (for example GED)

(q54:4)Some college, but degree not received or is in progress

(q54:5)Associate's degree (for example AA, AS)

(q54:6)Bachelor's degree (for example, BA, BS, AB)

(q54:7)Graduate degree (for example, master's, professional, doctorate)

(q54:8)Refused

-------------------------------------------------------------------------

Question: q55 - 1 (Single)

Text:

What is your current employment status?

(q55:1)Employed for wages

(q55:2)Self-employed

(q55:3)Out of work for 1 year or more

(q55:4)Out of work for less than 1 year

(q55:5)A homemaker

(q55:6)A student

(q55:7)Retired

(q55:8)Unable to work

(q55:9)Refused

-------------------------------------------------------------------------

Question: q56 - 1 (Single)

Text:

Generally speaking, do you think of yourself as a ...?

(q56:1)Democrat

(q56:2)Republican

(q56:3)Independent

(q56:4)Other

(q56:5)Don't know / not sure

(q56:6)Refused

-------------------------------------------------------------------------

Question: q57 - 8 (Numeric)

Text:

What is your annual household income from all sources- (if you are unsure of the exact amount, you may provide your best guess within $10,000). - Enter amount

-------------------------------------------------------------------------

Question: q57x - 1 (Multiple)

Text:

Exclusive Options: What is your annual household income from all sources- (if you are unsure of the exact amount, you may provide your best guess within $10,000).

(q57x:1)Don't know / not sure

(q57x:2)Refused

-------------------------------------------------------------------------

Question: q58 - 5 (Numeric)

Text:

What is your zipcode?

-------------------------------------------------------------------------

Question: q58x - 1 (Multiple)

Text:

Exclusive Options: What is your zipcode?

(q58x:1)Refused

-------------------------------------------------------------------------

Question: q59 - 1 (Single)

Text:

Would you be willing to participate in a future interview regarding vaccines? Participants will receive modest compensation for their time.

(q59:1)Yes

(q59:2)No

-------------------------------------------------------------------------

Question: q60 - 1 (Single)

Text:

Please provide a valid email address that we can contact you at for more information about a follow-up interview.

(q60:1)Yes

(q60:2)No

-------------------------------------------------------------------------

Question: q60oth1 - 50 (Open-end)

Text:

Please provide a valid email address that we can contact you at for more information about a follow-up interview. - Yes

-------------------------------------------------------------------------
